# IPF Respiratory Symptoms Management — Current Evidence

**DOI:** 10.3389/fmed.2022.917973

**Published:** 2022-07-28

**Authors:** Piotr Janowiak, Amelia Szymanowska-Narloch, Alicja Siemińska

**Affiliations:** Department of Pulmonology, Medical University of Gdańsk, Gdańsk, Poland

**Keywords:** breathlessness, dyspnea, cough, idiopathic pulmonary fibrosis (IPF), non-invasive ventilation (NIV), high flow nasal cannula (HFNC), ambulatory oxygen therapy (AOT), non-invasive positive pressure ventilation (NIPPV)

## Abstract

Idiopathic pulmonary fibrosis (IPF) is a progressive, chronic disease of the lungs which is characterized by heavy symptom burden, especially in the last year of life. Despite recently established anti-fibrotic treatment IPF prognosis is one of the worst among interstitial lung diseases. In this review available evidence regarding pharmacological and non-pharmacological management of the main IPF symptoms, dyspnea and cough, is presented.

## Introduction

Idiopathic pulmonary fibrosis (IPF) is the most frequent idiopathic interstitial pneumonia and one of the most frequent interstitial lung diseases (ILD) (1). It is a chronic, progressive disease of the lungs which has a distinct pattern, both radiologically and pathologically, of usual interstitial pneumonia (2). Despite recently established anti-fibrotic treatment IPF prognosis is one of the worst among ILDs with a mean survival of 2.5–5.0 years. Its course is characterized by heavy symptom burden especially in the last year of life (3, 4). Symptomatic treatment is an important aspect of palliative care which also addresses spiritual, social and psychological needs. Unfortunately, patients with IPF, as with other chronic lung diseases, are not referred to palliative care centers nearly as often as cancer patients. Furthermore, their symptomatic treatment is withhold partly due to the fear of opioids (5). Planning palliative care for IPF patients can also be hindered by heterogeneous course of the disease—loss of lung function might be gradual, rapid or suddenly accelerates because of a life-threatening acute exacerbation (3). This unpredictability is probably one of the reasons why the majority of IPF patients die in a hospital, subjected to life-prolonging procedures (6, 7). On the other hand, referring the IPF patient to palliative care too early, when the disease is mild, might worsen quality of life in short term, probably due to a worsening of depression or anxiety (8). Nonetheless, there is evidence that palliative care, compared to usual care, might improve respiratory symptoms and quality of life in patients with advanced IPF (9, 10).

The aim of this review is to present current evidence on symptomatic treatment of dyspnea and cough in IPF patients.

## Breathlessness—pathophysiology and treatment

Dyspnea in IPF results from both respiratory and circulatory limitations: reduced lung compliance, loss of lung volume, increased dead space ventilation, increased respiratory drive, gas exchange abnormalities and pulmonary hypertension (11). In turn, breathlessness treatment is a multifaceted process involving effective treatment of comorbidities, rehabilitation, pharmacological and oxygen treatment and non-invasive ventilation.

### Non-pharmacological treatment

#### Rehabilitation

Exertional hypoxemia, along with skeletal muscle dysfunction, restrictive ventilatory impairment and cardiovascular limitation, i.e., reduction in stroke volume, are responsible for exercise limitation in ILDs (12, 13). Exercise intolerance, in turn, is associated with reduced quality of life and increased mortality (14).

Cochrane review of 16 studies on pulmonary rehabilitation of ILD patients showed that this intervention can improve dyspnea and health-related quality of life, 6 min walking test (6MWT) distance and cardiopulmonary exercise test parameters, i.e., peak workload, peak oxygen uptake and maximum ventilation. Furthermore, evidence showed that improvements in dyspnea and SGRQ Impact score were sustained at 6–12 months. Sustained improvement affected not only dyspnea but also exercise capacity and health-related quality of life up to 12 months since rehabilitation program (15). Whether increases in 6MWT distance and peak oxygen uptake, both of which are predictors of IPF mortality, translate into prognosis improvement is unknown (16).

The positive effect of pulmonary rehabilitation on ILD patients is probably a result of repetitive chest expansion and stretching of the thoracic muscles what, in turn, translates into improvement of tidal volume. Increase in tidal volume leads then to improvement of peak oxygen uptake (16). Furthermore, it is suggested that rehabilitation improves peripheral oxygen extraction (17).

Rehabilitation of ILD patients is frequently complicated by desaturation which is why clinical supervision is essential for its safety and effectiveness (18). It is advised that supplemental oxygen should be used to maintain s_p_O_2_ ≥85% during exercise, if needed (18). To no surprise then, most of the studies (18 out of 21) included in the Cochrane review, were conducted in a supervised outpatient setting (15).

There is some conflicting evidence on time of referral to pulmonary rehabilitation for IPF patients. In studies by Kozu et al. and Holland et al. (19, 20) more advanced disease, i.e., lower mMRC score (20), lower forced vital capacity, greater exertional hypoxemia and higher right ventricular systolic pressure (19), predicted smaller improvement of 6MWT distance (20). It is worth underlining that such association was not evident for other than IPF ILD patients (19). On the other hand, study by Ryerson et al. showed that greater baseline 6MWT distance was associated with smaller 6MWT distance gain (21). It is author's view that the above should not discourage trial of pulmonary rehabilitation in advanced IPF patients.

#### Ambulatory and long term oxygen treatment

Exertional hypoxemia is one of the defining features of ILDs (12). Desaturation during 6MWT alone is an independent mortality and pulmonary hypertension risk factor (22–24). Furthermore, its severity in ILD is reported to be greater than in chronic obstructive pulmonary disease (COPD) (25). Nonetheless, studies failed to show unequivocal results of oxygen treatment in patients without significant hypoxemia at rest. Cochrane review of three crossover randomized controlled trials (RCT), performed in physiology laboratories, on 98 IPF patients altogether, failed to show any effect of short-term supplementary oxygen on exertional dyspnea (26). One of the studies showed increase in endurance time during constant load ergometry (27). None of them titrated oxygen to prevent desaturation, but used pre-determined fixed oxygen flow rate. Another systematic review, by Bell et al., incorporating 9 reports on short-term supplementary oxygen, showed similar results—no significant effect on dyspnea was detected while exercise capacity seemed to improve (28).

However, recently performed RCTs showed different results. A crossover RCT, by Dowman et al., on 11 patients with IPF, showed significant improvement of Borg dyspnea score and endurance time during cycle endurance test with oxygen supplementation where FiO2 (fraction of inspired oxygen) was set at 50%. It is noteworthy that Borg fatigue score did not improve (29). In another crossover RCT, on 20 fibrotic ILD patients, Schaeffer et al. showed that supplemental oxygen with FiO_2_ equal 60%, increased endurance time, reduced dyspnea and leg discomfort ratings (30). The most recent crossover RCT, AmbOx, the only study yet asserting effect of ambulatory oxygen treatment (AOT) on quality of life of fibrotic ILD patients, reported improvement of total K-BILD (King's Brief ILD questionnaire) scores and its breathlessness, activity and chest symptoms subdomains. However, this subjective improvement did not translate into increase of physical activity measured by biaxial accelerometer. It is worth underlining that each of the 76 AmbOx participants had flow rate of oxygen titrated during screening visit 6MWT to maintain s_p_O_2_ > 90%. Patients were then instructed to use their lightweight gas cylinders with the set flow during routine activities for 2 × 2 weeks (31). Authors reported that at the end of the trial 33% patients chose to discontinue oxygen treatment delivered *via* cylinders, however, those who experienced most dyspnea reduction were the most likely to continue. Younger age was also significantly predictive regarding the decision to continue AOT (31).

In light of the above national societies suggest a trial of AOT in patients with significant exertional desaturation, if there is evidence of benefit (32–34). One should take into consideration challenges connected with using the oxygen devices, especially outside home.

National guidelines are more unequivocal when ILD patients develop chronic hypoxemia at rest. Long term oxygen therapy (LTOT) is recommended even though the evidence is lacking (32–34). No RCTs were performed in this indication but three retrospective studies of which two did not include control group and none assessed effect of LTOT on breathlessness (28). Nonetheless, guidelines authors extrapolate evidence on survival benefit from COPD trials (32–34).

#### High flow nasal cannula

Compared to conventional oxygen therapy high flow nasal cannula (HFNC) provides oxygen at higher FiO_2_ (up to 100%) and at a higher flow, which matches patient's inspiratory demand and washes out CO_2_ from pharyngeal dead-space. Reduction of ventilatory dead-space might in turn improve the ventilation-perfusion inequality. High flow, up to 60 l/min, also generates a small amount of positive expiratory pressure. Furthermore, heating and humidifying of the respiratory mixture might reduce the metabolic cost of breathing (35). Laboratory studies show that HFNC might reduce work of breathing not only in healthy volunteers or COPD patients but in IPF patients as well (36, 37).

HFNC effectiveness in treating breathlessness was assessed in a study by Hui et al. on 30 advanced cancer patients, whose dyspnea intensity was ranked ≥3/10 despite supplemental oxygen. Patients received 2-h HFNC and 2-h NIPPV (non-invasive positive pressure ventilation), i.e., BiPAP, in a random sequence, both with FiO_2_ set at 100%. Dyspnea improved in both treatment arms, with no significant differences between, however, HFNC was better tolerated than BiPAP (38). Similar results were obtained by Koyauchi et al., who retrospectively assessed 84 ILD patients with do-not-intubate order and acute, hypoxic respiratory failure associated with ILD. Fifty-four patients used HFNC, 30—NIPPV. Temporary interruption of the therapy and discontinuation rates were significantly higher in the NIPPV group, whereas oral intake and ability to converse were significantly better in HFNC group. Three-day survival and in-hospital mortality did not differ significantly between the groups (39). Furthermore, HFNC compared to standard oxygen therapy seems to improve endurance time of IPF patients during constant-load exercise testing on cycloergometer in laboratory studies (40–42).

No studies so far have assessed domiciliary HFNC in ILD patients. However, recent two studies by Storgaard et al. in COPD patients with chronic hypoxemia show that HFNC used alongside LTOT might be of added benefit compared to LTOT alone (43, 44). In the first study, a RCT on 200 chronically hypoxemic COPD patients on LTOT, participants in HFNC group were instructed to use HFNC for 8 h daily, mainly during the night, as an add-on to LTOT, for at least 12 months. Seventeen percent of the participants discontinued HFNC. Thirty two percent used HFNC only during the day, 53% used it nightly, whereas the remaining 15% used HFNC both at night and day. Use of HFNC in conjunction with LTOT allowed for reduction of COPD acute exacerbation rate—which was the primary outcome. Furthermore, compared to LTOT alone, patients using HFNC additionally preserved their SGRQ score and 6MWT distance which dropped in the control group and reported significantly reduced mMRC score (43). Qualitative part of the study on 12 patients and 8 relatives showed that patients in the HFNC group found the device easy to use. Moreover, most patients reported that HFNC improved their sleep quality, despite the noise generated by the apparatus. In authors view this improvement was due to airway humidification and reduced work of breathing during sleep. Airway dryness, aggravated by LTOT, was reported by the patients as a significant reason for sleep interruption and awakening. Participants also reported a reduction in cough frequency (44).

Taking the above into consideration, HFNC seems as a viable option for oxygen delivery in IPF patients, although, high quality trials are needed.

#### Non-invasive ventilation

Respiratory exchange can be supported not only by conventional oxygen therapy or HFNC but also NIPPV. European Respiratory Society (ERS) and American Thoracic Society (ATS) guidelines suggest a trial of NIPPV in breathless patients in the setting of terminal condition (45). Available evidence encompasses two feasibility studies of RCT design in cancer patients (38, 46). Nava et al. randomized 200 end-stage cancer patients with solid tumors and acute respiratory failure to NIPPV (BiPAP) or oxygen. Only patients with PaO_2_/FiO_2_ ratio smaller than 250 were enrolled. Evident reversible causes of respiratory failure such as pulmonary edema were an exclusion factor. The study showed that NIPPV was significantly more effective in reducing dyspnea than conventional oxygen therapy but only in hypercapnic patients. Furthermore, 11% of NIPPV patients declined BiPAP due to poor tolerance (46). In a previously mentioned study, by Hui et al., NIPPV resulted in dyspnea reduction in cancer patients in a similar degree to HFNC, however it was significantly worse tolerated than HFNC (38).

The loss of lung compliance in IPF is associated with increased work of breathing and thus dyspnea. Offsetting the inspiratory burden by providing ventilation support could help treat breathlessness in IPF (11, 46). This would be especially true for patients in advanced stages of IPF who may develop hypercapnia—a sign of failing respiratory muscles unable to sustain the imposed load (11, 47). Hypercapnia in IPF could also be a sign of concomitant pleuroparenchymal fibroelastosis (PPFE), characterized by fibrosis involving the visceral pleura and subpleural parenchymal fibroelastosis. The resulting extrapulmonary restriction can in turn produce hypoventilation and hypercapnia (48, 49). Unfortunately, there are no studies which would assess effect of NIPPV on breathlessness in IPF patients. Data on NIPPV in ILD are limited to retrospective studies analyzing its effectiveness in treating acute respiratory failure, especially as means to avoid endotracheal intubation (50).

Dyspnea treatment summary, regarding AOT/LTOT, HFNC, and NIPPV, is presented in Table 1.

**Table 1:** Types of oxygen therapy and ventilatory support and their potential in treating breathlessness in IPF patients.

	**Mechanism of action**	**Evidence**	**Disadvantages**
AOT LTOT	- Improvement in neuro-mechanical uncoupling (74) - Lower neural respiratory drive (74) - Stimulation of upper airway receptors by gas flow (55) - Improved cardiovascular function and pulmonary hemodynamics (75) - Delayed lactate accumulation (76)	In patients with ILD and exertional hypoxemia: - Improvement of total K-BILD score and its subdomains i.e., breathlessness, activity and chest symptoms during routine activities of daily living (31) - Increase in cycle endurance time (28, 29) - Increase in CPET peak work capacity (28) - Increase in CPET peak oxygen uptake (28) - Reduction of Borg dyspnea score at the end of cycle exercise test and at the iso-time (29, 30) - Improved leg muscle oxygenation and reduction of fatigue (77) **Dyspnea was not assessed in LTOT studies in ILD patients** (28)	Portable oxygen systems • expensive, • may be hard to carry and difficult to use, • deliver limited amount of oxygen, • attract unwanted attention when used outside home Stationary oxygen systems • Risk of tripping over the tubing • Fire and burning hazard • Activity limited to the immediate surroundings of the oxygen system
HFNC	- Pharyngeal dead space washout (35) - Reduction of work of breathing (36, 37) - Matching patient's high inspiratory demand (35) - EPAP generation (up to 7.4 cm H_2_O) (78) - Lung compliance increase (35) - Upper airway resistance reduction (35) - Reduction of metabolic work associated with gas conditioning i.e., warming and humidifying (35) - Improvement of mucocilliary clearance (35)	Direct evidence i.e., based on studies involving ILD patients: - Increase in cycle endurance time (40–42) Indirect evidence: - Improvement of dyspnea in advanced cancer patients compared to oxygen therapy (38) - Improvement of dyspnea when used alongside LTOT in COPD patients compared to LTOT alone (43)	• Expensive • Only for stationary use • Requires high flow O2 source/sources (79) • Generates fair degree of noise (44)
NIPPV	- EPAP effects (80): *Lung compliance increase *Alveolar recruitment *Upper airway resistance reduction - IPAP effects (80): *Unloading of respiratory muscles *Tidal volume increase	*Indirect evidence:* - Improvement of dyspnea in advanced cancer patients compared to oxygen therapy (38), especially those with hypercapnic respiratory failure (46)	• Most expensive solution among discussed • Less comfortable than HFNC (38) • Doesn't allow for food intake and impedes speaking • Risk of face ulcerations secondary to tight-fitting masks • Patient might need help putting on the mask

6MWT, six-minute walking test; AOT, ambulatory oxygen therapy; CPET, cardiopulmonary exercise testing; EPAP, expiratory positive airway pressure; HFNC, high glow nasal cannulae; ILD, interstitial lung disease; IPAP, inspiratory positive airway pressure; IPF, idiopathic pulmonary fibrosis; K-BILD, King's Brief ILD questionnaire; LTOT, long term oxygen treatment; NIPPV, non-invasive positive pressure ventilation.

### Pharmacological treatment

#### Opioids

Pharmacological treatment of dyspnea mainly involves opioids which by acting upon their central and peripheral nervous system receptors can decrease anxiety, modulate central perception of dyspnea and reduce respiratory drive without significant changes in blood gases (51–55). Unfortunately, high quality RCTs of opioid effectiveness in treating ILD-related breathlessness are lacking. Available evidence includes retrospective, population based studies (4), open-label studies with oral opioids (56, 57) and RCTs with nebulized morphine (58–61). Only oral morphine studies showed its effectiveness in treating ILD-related dyspnea. However, one of them employed just a small group of 11 IPF patients (57), whereas the other studied a mixed group with a minority of ILD patients (*n* = 10; 12%). Nonetheless, guidelines do embrace oral morphine for IPF dyspnea treatment because of its proven effectiveness in chronic lung diseases in general (34). This recommendation is also backed up by data on opioid safety reported not only by retrospective (62) but also prospective studies (63).

## Cough

In IPF cough is one of the most frequent symptoms reported by 50–80% of patients (64, 65). Though it is typically described as dry (66), more than half of patients might expectorate sputum (64). Furthermore, cough was found to be an independent predictor of disease progression (64). Chronic cough in IPF can weigh heavily on quality of life by interrupting sleep, limiting speech or by causing significant desaturation, musculoskeletal pain and urinary incontinence. It is to no surprise then that cough might limit social interactions (67).

Mechanism of chronic cough induced by IPF is not precisely understood. It is assumed that increased cough reflex sensitivity, which is in complex interplay with frequent IPF co-morbidities—gastroesophageal reflux disease and obstructive sleep apnea, is involved. This increased sensitivity might be a result of increased traction forces impacting the function of stretch receptors. Other possible mechanisms involve destruction of inhibitory nerves by fibrosis or upregulation of vagal sensory fibers (66, 67). A significant role of stretch receptors in pathogenesis of IPF cough seems to be confirmed by results of the study by Jones et al. (68). Authors found that IPF patients, compared with healthy non-smoking controls, were significantly more susceptible to induction of non-productive cough by mechanical percussion of the chest wall especially when percussor was applied to the posterior lung base (68). As pointed out by van Manen, this correlates with the clinical finding that vibration caused by talking or coughing starts a self-perpetuating cough cycle (67).

Taking the above into consideration, a trial of treatment of IPF cough with neuromodulator i.e., gabapentin, as in idiopathic chronic cough, is recommended (69). Its effectiveness in idiopathic chronic cough was assessed in a RCT on 62 patients, who experienced significant improvement of cough-specific quality of life after 8 weeks of treatment. However, 10 patients, 31% of the gabapentin group, reported side effects with nausea and fatigue as the most common (70). Speech therapy, especially combined with pregabalin is also recommended (66, 69). If the above fails, guidelines suggest a trial of opioids (69). Nonetheless, this recommendation is based only on one RCT, of small-dose, slow-releasing morphine in 27 patients with idiopathic chronic cough (71).

Two other drugs have been trialed in IPF cough and showed efficiency. In a crossover RCT by Horton et al., thalidomide, a potent immunomodulatory drug, significantly improved cough-related quality of life in 20 patients with IPF during 12 weeks of treatment. However, 77% of patients reported side-effects: constipation, dizziness, malaise, anorexia and asymptomatic bradycardia (72). In light of these results thalidomide was not recommended by CHEST Expert Cough Panel experts in treatment of cough in IPF. Small size of the study population, side effects, prescription barriers and cost were among factors influencing this decision (69). In a crossover RCT by Birring et al., a novel formulation of sodium cromoglicate delivered by mesh nebulizer reduced cough frequency but did not improve cough-specific quality of life or cough severity in 24 IPF patients after 2 weeks of treatment (73).

## Conclusions

Evidence for different interventions in symptomatic treatment of IPF patients is lacking. Consequently, IPF guidelines often base their recommendations on trials conducted in other chronic lung diseases. High quality trials are needed to verify efficiency of guidelines-compliant pharmacological treatment of cough and breathlessness in IPF patients. Positive results of AOT, NIPPV, and HFNC in treatment of breathlessness in other lung diseases should encourage similar studies in IPF patients as well.

## Author contributions

PJ wrote the first draft of the manuscript. All authors contributed to conception of the review. All authors contributed to manuscript revision, read, and approved the submitted version.

## Conflict of interest

The authors declare that the research was conducted in the absence of any commercial or financial relationships that could be construed as a potential conflict of interest.

## Publisher's note

All claims expressed in this article are solely those of the authors and do not necessarily represent those of their affiliated organizations, or those of the publisher, the editors and the reviewers. Any product that may be evaluated in this article, or claim that may be made by its manufacturer, is not guaranteed or endorsed by the publisher.
